# Treatment outcome and its associated factors among HIV-MTB co-infected patients in Sichuan, China: A retrospective study

**DOI:** 10.1097/MD.0000000000032006

**Published:** 2022-12-02

**Authors:** Ni Yang, Chuang Chen, Jinge He, Jing Li, Yin Zhong

**Affiliations:** a Sichuan Center for Disease Control and Prevention, Chengdu, Sichuan, China.

**Keywords:** China, HIV-MTB co-infection, surveillance, treatment outcome

## Abstract

Human immunodeficiency virus (HIV)-*Mycobacterium tuberculosis* (MTB) co-infection has become a pressing global public health problem. Although tuberculosis (TB) is both treatable and curable, it has been exacerbated by the HIV/acquired immune deficiency syndrome (AIDS) epidemic. HIV-MTB co-infected patients have a variety of disease-specific, and treatment-related factors that can adversely affect their treatment outcomes. This study was conducted to assess the outcomes of TB treatment and its associated factors among HIV-MTB co-infected patients in Sichuan, Southwest China. A retrospective study was performed on HIV-MTB co-infected patients who were diagnosed and registered in TB designated hospitals in Sichuan from January 1, 2016, to December 31, 2020. Data were collected from patients’ electronic medical records regarding their demographic, clinical, and social support information, and categorical data, such as sex, were reported using numbers and percentages. *χ*^2^ and *t*-tests were conducted to compare groups in relation to different levels of medical institutions. A binary logistic regression model was used to identify the factors associated with unsuccessful TB treatment outcomes. For logistic regression analysis performed using an *α* of 0.05, odds ratios (ORs) and corresponding 95% confidence intervals (CIs) were calculated for various risk factors. A total of 3677 registered HIV-MTB co-infected patients were enrolled. After adjusting for other variables, male, advanced age, receiving TB treatment at the municipal medical institution, being diagnosed with external pulmonary TB, referral or tracing, being sputum smear positive, not initiating antiretroviral therapy (ART) and not using fixed-dose combinations were the main risk factors for treatment failure of HIV-MTB co-infected patients in Sichuan province. Sex, age, hospital level, patient source, other diagnostic factors (e.g., sputum smear results, anatomical site of TB), and factors of therapeutic schemes (e.g., antiretroviral therapy, fixed-dose combinations) may serve as risk factors to estimate the likely treatment outcome of HIV-TB co-infection.

## 1. Introduction

Tuberculosis remains a public health concern among people living with human immunodeficiency virus (HIV)/acquired immune deficiency syndrome (AIDS) (PLH), resulting in increased morbidity and mortality.^[1]^ Among HIV-related diseases, tuberculosis (TB) accounts for 26% of AIDS-related deaths,^[2]^ almost (99%) of which occur in developing countries.^[3]^ There were an estimated additional 208,000 TB deaths (range, 177,000–242,000) among HIV-positive people.^[4]^ Although the deaths of TB patients among PLH account for only 15% of the total TB deaths, in some countries with a high burden of TB, such as South Africa, efforts to control TB are overwhelmed by the rising number of TB cases occurring in parallel with the HIV/AIDS epidemic.^[5]^ The HIV epidemic continues to fuel a TB epidemic.

Both TB and HIV can drastically affect the host immune system because they can evade immune surveillance and clearance; however, the underlying mechanisms are not fully understood.^[3]^ In an individual host, 2 pathogens, Mycobacterium tuberculosis (*M tuberculosis*) and HIV, accelerate the deterioration of immunological functions and result in premature death if left untreated.^[6]^

Even in this circumstance of the introduction of high active antiretroviral therapy, TB infection still places a great burden on PLH in Chinese mainland, a developing country.^[7]^ As reported in a meta-analysis in China, the overall prevalence of TB infection among PLH was estimated to be 7.2% (range: 4.2–12.3%) and was even higher (22.8%) in AIDS patients.^[8]^ According to the World Health Organization (WHO) policies, China annually provides TB screening for those diagnosed with HIV/AIDS and HIV testing for those diagnosed with TB in regions with high HIV/AIDS epidemics.

Sichuan Province is characterized by the largest population living with HIV/AIDS in Southwest China. Owing to its remote geographical position and the limitations of local medical services, Sichuan has a high incidence of TB (approximately 100 cases per 100,000 people),^[9]^ with the second highest TB caseload among the provinces in China.^[10]^ The HIV testing coverage and HIV-positive case detection rates in Sichuan were estimated to be approximately 76% and 0.9%, respectively, as revealed in the TB report of China 2019 from the Center of Disease Control and Prevention of China. Nonetheless, another investigation in Sichuan showed that HIV testing was not universally available for outpatient TB cases; only 29.5% (3227/10,946) of cases had HIV test results.^[11]^ The prevalence of HIV infection among patients with TB is high in locations where the greatest increase in TB cases has occurred. A cross-sectional study conducted in Sichuan, Guangxi, and Henan revealed that among TB patients, 3.3% were HIV-positive.^[12]^

Despite the latest treatment outcome data showing a TB treatment success rate of 84% and antiretroviral therapy (ART) initiation rate of 88% for those with HIV-MTB co-infection in a 2015 cohort in China,^[13]^ the mortality of those with HIV-MTB co-infection remains frustrating at 36.5% in southern China according to a recent 5-year-cohort study.^[14]^ Various studies have reported an association between unsuccessful TB treatment and risk factors. However, inconsistent findings from previous studies have made it difficult to assess whether reported factors are applicable to the local environment. In addition, little is known about the factors related to unsuccessful TB treatment outcomes in HIV-MTB co-infected patients who received ART initiation and anti-TB therapy. Consequently, identifying the impact of epidemiological and clinical characteristics on unsuccessful treatment outcomes in patients with HIV-MTB co-infection would provide important insights and a scientific basis for HIV-MTB co-infection prevention, control, diagnosis, and treatment in the local healthcare system.

## 2. Materials and Methods

### 2.1. Design, setting, and population

#### 2.1.1. Design and setting.

This study was conducted in Sichuan, Southwest China. This retrospective study used data from the Chinese National TB Surveillance System (NTSS) database. Following international guidelines, coordinated registration and care systems were initiated between the HIV and TB programs in 2010. In line with WHO policies, care services such as antiretroviral therapy, anti-tubercular treatments, isoniazid preventive therapy, and infection control are now coordinated with the Chinese national HIV and TB programs.

### 2.2. Population

Patient data were considered for this study if they were diagnosed with HIV/AIDS and MTB (including pulmonary TB and extra-pulmonary TB) and were reported and registered in NTSS at all designated TB or HIV medical institutions in Sichuan over a period of 5 years (2016–2020).

A diagnosis of MTB was made based on the combined evaluation of the clinical, radiological, histopathological, and laboratory features of the patients in accordance with the protocol established by the National Tuberculosis Prevention and Control Program (2008): sample smears/cultures positive or sample smears/cultures negative but met all 3 of the following clinical criteria:

1. Symptoms consistent with TB;
2. chest X-ray suggestive of TB; and
3. positive anti-TB Rx response.

A patient was considered HIV-infected when a screening test by enzyme-linked immunosorbent assay tested positive, HIV strains were isolated from the blood, or both results of the screening test were positive at different times. For TB patients, hospitals provide directly observed therapy (DOT) in both adult and pediatric age groups with free drugs like 2 months intensive phase with rifampicin, isoniazid, pyrazinamide, and ethambutol (2HRZE) and 4 months continuation phase with rifampicin and isoniazid (4RH).

### 2.3. Variable measurement

Sociodemographic and clinical variables related to the study objectives were obtained from the TB treatment registry. The former included age, sex (male or female), ethnicity (Han, the other minority), occupation (unemployed, worker, farmer, and others), level of medical institution (prefecture/city-level, county/district-level); the latter included the results of the initial sputum smear test (positive, negative/not performed), type of case finding (clinic visit due to symptoms, referral/tracing, and others [contact examination/health examination/recommendation/other]), patient type (new cases, relapse/return after loss to follow-up), HIV antibody test results (known positive, newly confirmed positive), site of TB (pulmonary, external pulmonary), rifampicin-resistant TB (sensitivity, resistance, not performed), ART (yes, no), and fixed-dose combinations (yes, no). In addition to the standard outcome definitions, we classified the final TB treatment outcome as successful (cure or complete treatment) or unsuccessful (death, failure, adverse events, loss to follow-up, or not evaluated).

### 2.4. Data analysis and statistics

The abstracted data were entered into Excel 2010 and exported to SPSS, version 17.0 (IBM SPSS Inc, Armonk). Chi-square tests and t-tests were conducted to compare groups in relation to different levels of medical institutions. Descriptive statistics of the variables of interest are expressed as absolute and relative frequencies or mean ± standard deviation (SI). The association measures for each variable category were estimated using univariate and multivariate logistic regression models. The outcome variable was whether the patient experienced an unsuccessful outcome after the treatment. A logistic regression analysis was performed using an *α* value of 0.05. Various risk factors were calculated using odds ratios (ORs) at *P* < .05, and 95% confidence intervals (CIs). The goodness of fit of the model was tested using the Hosmer–Lemeshow test.

## 3. Results

There were 230,794 cases of TB cases reported and registered in Sichuan between 2016 and 2020, of which 3677 (1.59%) were HIV/AIDS co-infected and were included in the final analysis.

Table 1 shows the sociodemographic distribution and outcomes of HIV-MTB co-infected patients at the medical institution level in Sichuan from 2016 to 2020. The majority of the patients were from county-level hospitals: 3431(93.31%), male 2977 (80.96%), Han ethnicity 2055 (55.89%), farmers 3024 (82.24%), and most TB patients with HIV co-infection aged between 33 and 53 years (76.3%). Of these, 3273 (89.01%) had successful outcomes and 404 (10.99%) had unfavorable outcomes. Among those with successful outcomes, 862 (23.44%) were cured, and 2411 (65.57%) were treated. Of these, 243 (6.61%) patients died, 19 (0.52%) experienced treatment failure, 15 (0.41%) experienced adverse events, 24 (0.65%) were lost to follow-up, and 103 (2.8%) were not evaluated. Overall, the prevalence of unsuccessful (2) treatment outcomes was higher in HIV-MTB co-infected patients from city-level institutes than from county-level institutes (22.36% vs. 10.17%).

**Table 1:** Distribution of socio-demographic and treatment outcome of HIV-MTB co-infection cases by level of medical institution. Sichuan, China, 2016–2020.

Characteristics		Level of medical institution		*P*-value
	N (%)	County-level N (%)	City-level N (%)	
**Total (n**)	3677	3431 (93.31)	246 (6.69)	
**Gender**				.327*
Female	700 (19.04)	659 (19.21)	41 (16.67)	
Male	2977 (80.96)	2772 (80.79)	205 (83.33)	
**Age, mean ± SD**	43.66 ± 14.29	43.38 ± 14.20	47.68 ± 14.82	.000†
**Ethnicity**				.000*
Han	2055 (55.89)	1847 (53.83)	208 (84.55)	
The other minority	1622 (44.11)	1584 (46.17)	38 (15.45)	
**Occupation**				.000*
Unemployed	451 (12.27)	398 (11.60)	53 (21.54)	
Worker	89 (2.42)	79 (2.30)	10 (4.07)	
Farmer	3024 (82.24)	2849 (83.04)	175 (71.14)	
The others	113 (3.07)	105 (3.06)	8 (3.25)	
**Outcome**				
**Successful outcome**	3273 (89.01)	3082 (89.83)	191 (77.64)	
Cured	862 (23.44)	813 (23.70)	49 (19.92)	.000*
Treatment complete	2411 (65.57)	2269 (66.13)	142 (57.72)	
**Unsuccessful outcome**	404 (10.99)	349 (10.17)	55 (22.36)	
Died	243 (6.61)	213 (6.21)	30 (12.20)	.000*
Failure	19 (0.52)	19 (0.55)	0 (0)	
Lost to follow-up	24 (0.65)	24 (0.70)	0 (0)	
Adverse event	15 (0.41)	15 (0.44)	0 (0)	
Not evaluated	103 (2.80)	78 (2.27)	25 (10.16)	

HIV = human immunodeficiency virus, MTB = *Mycobacterium tuberculosis*, SD = standard deviation.

* Chi-square test.

† T-test.

The univariate logistic regression model (Table 2) revealed that sex, age, level of medical institution, type of case finding, TB form, sputum smear examination results, patient type, antiretroviral therapy, and fixed-dose combinations were significantly associated with unsuccessful treatment outcomes. Unsuccessful treatment outcomes were more likely to occur in male patients than in female patients (OR = 1.39, 95% CI: 1.05–1.86, *P* = .024). The odds of unsuccessful treatment for patients from city-level hospitals were 2.5 times higher than those from county-level hospitals. Increased age was a statistically significant predictor of unfavorable treatment outcomes among HIV-MTB co-infected patients (OR = 1.01, 95% CI: 1.00–1.02, *P* = .002). In addition, external pulmonary TB patients had a 2.3 times higher chance of developing unfavorable outcomes than pulmonary TB patients did. Relapsed or returned treatment after being lost to follow-up had increased odds of unfavorable outcomes compared to new patients (OR = 1.59, 95% CI: 1.11–2.28, *P* = .012). Patients who visited the clinic due to symptoms (OR = 2.10, 95% CI: 1.21–3.66, *P* = .009) or via referral and tracing mechanisms (OR = 2.41, 95% CI: 1.37–4.24, *P* = .002) had higher odds of unsuccessful outcomes than the others (health/contact examination/recommendation/other). Moreover, the odds of unsuccessful treatment were 1.6 times higher in smear-positive patients than in smear-negative patients and 1.4 times higher in antiretroviral therapy-free patients than in antiretroviral therapy-treated patients. Patients receiving TB treatment without fixed-dose combinations (OR = 1.44, 95% CI: 1.17–1.77, *P* = .001) had a significantly increased risk of unsuccessful treatment outcomes than those with fixed-dose combinations.

**Table 2:** Association between demographic/clinical characteristics and treatment outcome among HIV-MTB co-infection patients in Sichuan, 2016–2020.

Characteristics	Successful outcome, N = 3273 (%)	Unsuccessful outcome, N = 404 (%)	OR (95% CI)	*P*-value
**Gender**				
Female	640 (91.43)	60 (8.57)	Ref	
Male	2633 (88.44)	344 (11.56)	1.394 (1.045, 1.858)	.024
**Age**			1.011 (1.004, 1.019)	.002
**Ethnicity**				
The other minority	1457 (89.83)	165 (10.17)	Ref	
Han	1816 (88.37)	239 (11.63)	1.162 (0.942, 1.434)	.161
**Occupation**				
Unemployed	405 (89.80)	46 (10.20)	Ref	
Worker	83 (93.26)	6 (6.74)	0.636 (0.263, 1.539)	.316
Farmer	2678 (88.56)	346 (11.44)	1.138 (0.822, 1.574)	.437
The others	107 (94.69)	6 (5.31)	0.494 (0.205, 1.187)	.115
**Medical institute**				
County-level	3082 (89.83)	349 (10.17)	Ref	
City-level	191 (77.64)	55 (22.36)	2.543 (1.847, 3.501)	.000
**Type of case finding**				
The others (contact examination/health examination/recommendation/other)	240 (94.49)	14 (5.51)	Ref	
Referral/tracing	1048 (87.70)	147 (12.30)	2.405 (1.365, 4.234)	.002
Clinic visit due to symptoms	1985 (89.09)	243 (10.91)	2.099 (1.205, 3.656)	.009
**Site of TB**				
Pulmonary TB	3139 (89.51)	368 (10.49)	Ref	
Extra pulmonary TB		36 (21.18)	2.292 (1.562, 3.363)	.000
**Patient type**				
New case	3067 (89.36)	365 (10.64)	Ref	
Relapse or return treatment after lost to follow-up	206 (84.08)	39 (15.92)	1.591 (1.111, 2.278)	.012
**Initial sputum smear**				
Negative/not performed	2263 (90.59)	235 (9.41)	Ref	
Positive	1010 (85.67)	169 (14.33)	1.611 (1.305, 1.990)	.000
**HIV antibody test result**				
Known positive	2550 (89.51)	299 (10.49)	Ref	
New confirmed positive	723 (87.32)	105 (12.68)	1.239 (0.977, 1.570)	.077
**Antiretroviral therapy**				
Yes	1399 (90.90)	140 (9.10)	Ref	
No	1874 (87.65)	264 (12.35)	1.408 (1.134, 1.748)	.002
**Rifampicin-resistant TB**				
Sensitivity	38 (88.37)	5 (11.63)	Ref	
Resistance	731 (88.61)	94 (11.39)	0.977 (0.375, 2.544)	.962
Not performed	2504 (89.14)	305 (10.86)	0.926 (0.362, 2.370)	.872
**Fixed-dose combinations**				
Yes	1913 (90.53)	200 (9.47)	Ref	
No	1360 (86.96)	204 (13.04)	1.435 (1.166, 1.765)	.001

CI = confidential interval, HIV = human immunodeficiency virus, MTB = *Mycobacterium tuberculosis*, OR = odds ratio, TB = tuberculosis.

Multivariate analysis of the characteristics associated with unsuccessful treatment is shown in Table 3. After adjusting for other variables, the final model identified that sex, age, level of medical institution, type of case-finding, site of TB, result of sputum smear examination, antiretroviral therapy, and fixed-dose combinations were significantly associated with unsuccessful treatment in this group. The odds of unsuccessful outcomes were significantly higher among male patients with HIV-MTB than among female patients (adjusted odds ratio [AOR] = 1.36, 95% CI: 1.01–1.82, *P* = .041). For each 1-year increase in age, the odds of unsuccessful treatment increased by 0.9%. Meanwhile, the odds (AOR = 2.37, 95% CI: 1.70–3.29, *P* = .000) were higher for patients who sought medical aid at the city-level medical institution than those at the county-level medical institution. Patients who visited the clinic via referral or tracing were 2.2 times more likely to develop an unfavorable outcome than those who visited the clinic through other means, such as contact examination, health examination, and recommendation. External pulmonary TB patients with HIV co-infection were more likely (AOR = 1.98, 95% CI: 1.33–2.95, *P* = .001) to develop unsuccessful outcomes than simple pulmonary TB patients co-infected with HIV/AIDS. Sputum smear-positive patients had increased odds of unsuccessful outcomes (AOR = 1.52, 95% CI: 1.22–1.89, *P* = .000) compared with sputum smear-negative/non-performed HIV-MTB patients. The odds of unfavorable outcomes were 1.4 times higher in patients who did not receive ART than in those who initiated antiretroviral therapy. Patients without fixed-dose combinations had a statistically significant increase in the chance of developing unsuccessful outcomes compared to those who used fixed-dose combinations (AOR = 1.39, 95% CI: 1.12–1.73, *P* = .003).

**Table 3:** Socio-demographic and clinical factors associated with treatment outcome in HIV-MTB co-infection patients. Sichuan, 2016–2020.

Characteristics	AOR (95% CI)	*P*-value
**Gender**		
Female	Ref	
Male	1.36 (1.01, 1.82)	.041
**Age**	1.01 (1.00, 1.02)	.015
**Medical institution**		
County-level	Ref	
City-level	2.37 (1.70, 3.29)	.000
**Type of case finding**		
The others (contact examination/health examination/recommend/other)	Ref	
Referral/tracing	2.19 (1.24, 3.90)	.007
Visit clinic due to symptoms	1.71 (0.97, 3.02)	.064
**Site of TB**		
Pulmonary TB	Ref	
External pulmonary TB	1.98 (1.33, 2.95)	.001
**Initial sputum smear**		
Negative/Not performed	Ref	
Positive	1.52 (1.22, 1.89)	.000
**Antiretroviral therapy**		
Yes	Ref	
No	1.39 (1.12, 1.73)	.003
**Fixed-dose combinations**		
Yes	Ref	
No	1.39 (1.12, 1.73)	.003

AOR = adjusted odds ratio, CI = confidential interval, HIV = human immunodeficiency virus, MTB = mycobacterium tuberculosis, TB = tuberculosis.

## 4. Discussion

Tuberculosis and HIV co-infection remain among the most serious public health challenges in Southwest China. In this study, we found NTSS an overall TB treatment success rate of 89% for 3677 TB patients living with HIV in 5 years (2016–2020), which is higher than the national treatment success rate of 76.0% in 2018, which is in agreement with studies conducted in Asian countries such as Vietnam (74.0%),^[15]^ India (80.0%),^[16]^ and Thailand (74.3%).^[17]^

Based on their self-descriptions, the majority of TB patients living with HIV were male, which is in line with the findings of other studies.^[18]^ Furthermore, males were more at risk for an unsuccessful treatment outcome, which was in agreement with the study by Engelbrecht.^[19]^ The higher rates of unsuccessful treatment outcomes in men may be due to high-risk behaviors, such as alcohol use, smoking, and illicit drug abuse.

The majority (76.3%) of the HIV-MTB patients were in the age group 33 to 53 years. The rate of unsuccessful treatment was significantly associated with advanced age. Several studies have reported that advanced age is associated with unsuccessful treatment.^[20,21]^ Not surprisingly, advanced age is a risk factor for mortality in patients with TB with or without HIV infection.

In this study, the medical institution where an HIV-MTB co-infection patient sought medical aid was found to be associated with unsuccessful outcomes. The odds of an unsuccessful TB treatment outcome were 2.367 times higher for patients seeking medical assistance at city-level hospitals than at county-level institutes. This is similar to an earlier study conducted in Zambia^[22]^ and a study done in northern Ethiopia.^[23]^ HIV-MTB co-infected patients who visit county-level medical institutions mostly seek medical advice after they have high-risk behaviors that may lead to HIV infection so that they can know their HIV infection status in a timely manner and be diagnosed early. They can then receive antiviral and anti-TB treatments. In contrast, patients who choose to seek treatment at city-level medical institutions might be because their immune system has already been seriously harmed, and opportunistic infections or AIDS-related diseases are detected when they seek medical treatment, thus affecting the treatment effect of patients with dual infections. This area needs to be studied in the future to determine which risk factors exist in different locations that may put people at a higher risk of unfavorable outcomes in TB treatment.

We also found that identification of patients through referral or tracing was a predictor of unsuccessful treatment outcomes. This is partly because people who were identified with TB through contact or health examination, recommendations, and other methods are more likely to be compliant with care and to pay attention to their own health status than those identified through referral or tracing; therefore, they are more likely to have successful treatment outcomes. Our study sheds light on a neglected issue that requires attention, namely, that identifying patients earlier through voluntary counseling and examining programs is essential to help reduce TB mortality in co-infected patients. This result is consistent with that reported by Teasdale^[24]^ and Jacobson.^[25]^

Khan et al showed that external pulmonary TB-HIV is a risk factor for poor TB outcomes.^[26]^ This is consistent with the current study, which found that external pulmonary TB-HIV infection is a 1.98-fold risk factor for unsuccessful outcomes. Another study showed that external pulmonary TB is more common in the HIV-associated TB population, becomes increasingly prevalent with progressive immunodeficiency, and contributes to unfavorable treatment outcomes.^[27]^

The chance of unsuccessful outcomes was higher among HIV-MTB co-infected patients with smear-positive sputum samples. Similar findings have been reported in other studies conducted in South India^[28]^ and eastern Ethiopia.^[29]^ In contrast, a study conducted by Mizan Tepi^[30]^ demonstrated that HIV-MTB co-infected patients with smear-positive pulmonary TB had a higher chance of successful TB treatment outcomes.

Half (58.14%) of the patients did not receive ART at the time of initiation of TB treatment. An example of the difficulties faced by this group is that in some Brazilian cities, patients can receive high active ART but not food vouchers or transportation subsidies. The absence of these 2 services makes successful treatment virtually impossible.^[31]^

Not using fixed-dose combinations was significantly associated with unsuccessful treatment outcomes in patients with HIV co-infection. One of the factors for unsuccessful treatment outcomes might be the load of TB drugs and HIV-drug interactions. HIV-MTB co-infected patients use more drugs and are more likely to experience more drug side effects than patients taking HIV ART or anti-TB drugs only. Other studies have also reported that drug interactions have more side effects on the treatment outcomes of HIV-MTB co-infection.^[32]^

Surprisingly, rifampicin-resistant HIV-MTB, HIV antibody test results, and relapse or return due to loss to follow-up were not significantly associated with unsuccessful treatment outcomes in multivariate hazard ratio analysis of treatment outcomes. Similarly, a study conducted in Nigeria found no association between new and relapsed patients.^[33]^

A major strength of this study is that the large sample size allowed us to identify the independent risk factors for unsuccessful treatment outcomes. In addition, the study was carried out under routine conditions, meaning that the data were representative of the real situation; thus, the findings are generalizable to most of the populous provinces in China, which account for a large proportion of pulmonary TB patients with HIV co-infection in the country.

Apart from these significant findings, this study also has several limitations that are characteristic of retrospective studies. All data were dependent on paper registers kept in the hospital and health clinics, which means that it was neither feasible to correct any obvious errors in the data nor could missing data be captured. This excluded other important variables that could have influenced the outcome of TB treatment in HIV-MTB co-infected patients. For example, cluster of differentiation 4 T cell count, viral load, education level, smoking, and other co-infections. Although we attempted to locate missing information from the HIV/AIDS surveillance system this was not always possible because there was not yet a publicly available list of patients to match the TB surveillance system. Therefore, this missing information may have biased risk estimates. Bias is always a concern in secondary data sources. The NTSS database has served as a reliable source for several other population-based studies in China.

## 5. Conclusion

In this study, the overall success rate of TB treatment was almost in line with the WHO target of 90%. Age, sex, facility level, type of case finding, anatomic site of TB result of sputum examination, ART initiation, and fixed-dose combinations use were predictors of unsuccessful TB treatment outcomes in HIV-MTB co-infected patients in Sichuan. These findings have broad policy implications. Providing better social support to patients could be key to improving treatment outcomes. Therefore, we would like to propose the following recommendations:

(1) HIV-MTB co-infected patients who did not receive ART or who did not use fixed-dose combinations due to side effects should be encouraged individually for counseling in the directly observed therapy (DOT) clinic on the side effects of anti-TB/ anti-virus drugs and the risk associated with disrupting TB treatment or not initiating antiretroviral therapy.
(2) There is a pressing need to strengthen cooperation between TB and HIV control programs as well as communication between patients and health care workers. Specific initiatives could include incorporating HIV/AIDS counseling and screening for HIV among TB patients into current TB control program assessments, which could achieve better TB treatment outcomes in southwest China.

## Acknowledgments

The authors would like to thank Dr Zhang and Dr Kang for linguistic assistance.

## Author contributions

Ni Yang designed the study, performed statistical analyses and data interpretation, and wrote the main manuscript. Jing Li and Yin Zhong contributed to the data acquisition. Chuang Chen contributed to manuscript revisions. Jinge He supervised this study. All the authors have reviewed the manuscript.

**Data curation**: Yin Zhong, Jing Li.

**Supervision**: Chuang Chen.

**Validation**: Jinge He.

**Writing – original draft**: Ni Yang.

**Writing – review & editing:** Ni Yang.
